# Biosynthesis of *C*-nucleoside antibiotics in actinobacteria: recent advances and future developments

**DOI:** 10.1186/s12934-021-01722-z

**Published:** 2022-01-04

**Authors:** Meng Zhang, Liyuan Kong, Rong Gong, Marianna Iorio, Stefano Donadio, Zixin Deng, Margherita Sosio, Wenqing Chen

**Affiliations:** 1 Key Laboratory of Combinatorial Biosynthesis and Drug Discovery, Ministry of Education, and School of Pharmaceutical Sciences, Wuhan University, Wuhan, 430071 China; 2 State Key Laboratory of Microbial Metabolism, and School of Life Sciences and Biotechnology, Shanghai Jiao Tong University, Shanghai, 200240 China; 3 Naicons Srl, Viale Ortles 22/4, 20139 Milan Italy

**Keywords:** Natural product, *C*-nucleoside antibiotics, Biosynthesis, Genome mining, Synthetic biology

## Abstract

Epidemic diseases and antibiotic resistance are urgent threats to global health, and human is confronted with an unprecedented dilemma to conquer them by expediting development of new natural product related drugs. *C*-nucleoside antibiotics, a remarkable group of microbial natural products with diverse biological activities, feature a heterocycle base linked with a ribosyl moiety via an unusual *C*-glycosidic bond, and have played significant roles in healthcare and for plant protection. Elucidating how nature biosynthesizes such a group of antibiotics has provided the basis for engineered biosynthesis as well as targeted genome mining of more *C*-nucleoside antibiotics towards improved properties. In this review, we mainly summarize the recent advances on the biosynthesis of *C*-nucleoside antibiotics, and we also tentatively discuss the future developments on rationally accessing *C*-nucleoside diversities in a more efficient and economical way via synthetic biology strategies.

## Introduction

Nucleoside antibiotics play significant roles in mammalian healthcare and for plant protection against pathogen-induced infections [1], and they are usually synthesized by sequential modifications of nucleosides or nucleotides of primary origin to render the intricate molecules [2]. Nucleoside antibiotics, on the basis of the linkage by glycosidic bond, can be generally classified into two main categories, either *N*- or *C*-nucleosides. Normally, *N*-nucleosides are structurally unstable and susceptible to cleavage of the glycosidic bond with loss of bioactivity, which has raised considerable research interests in the past decades towards the development of *C*-nucleoside derived drugs [3]. Actually, nature, as a great chemist, has developed the great capability of creating the *C*-nucleosides prior to the synthesis of artificial molecules, and pseudouridine (Ψ) is the first naturally-occurring *C*-nucleoside found abundantly in tRNA [4]. Since then, several *C*-nucleoside antibiotics have been successively isolated in the past decades.

**Fig. 1:**
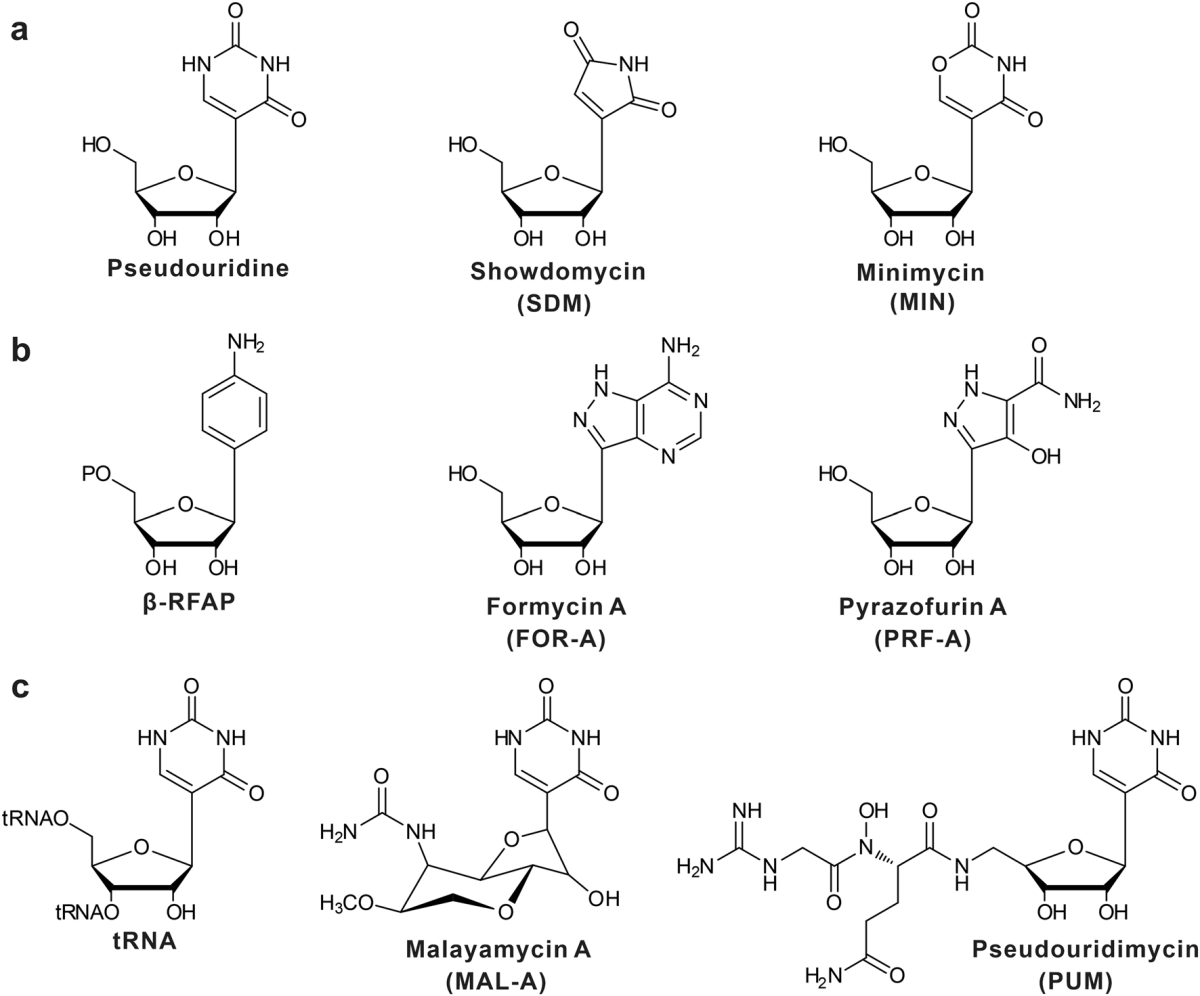
Chemical structures of *C-*nucleoside antibiotics featured in this review. **a** Structures of showdomycin (SDM), minimycin (MIN) and related compounds; this subgroup employs a YeiN-like strategy for *C*-glycosidic bond construction. **b** Structures of formycin A (FOR-A), pyrazofurin A (PRF-A) and structural analogs; for this subgroup, β-RFAP (β-ribofuranosyl-aminobenzene 5′-phosphate) synthase-like enzymes govern *C*-nucleoside scaffold construction in the biosynthesis of FOR-A and PRF-A. **c** Structures of tRNA, malayamycin A (MAL-A), and pseudouridimycin (PUM); the tRNA pseudouridylate synthases (TruD-like) are responsible for *C*-glycosidic bond formation in MAL-A and PUM biosynthesis

**Fig. 2:**
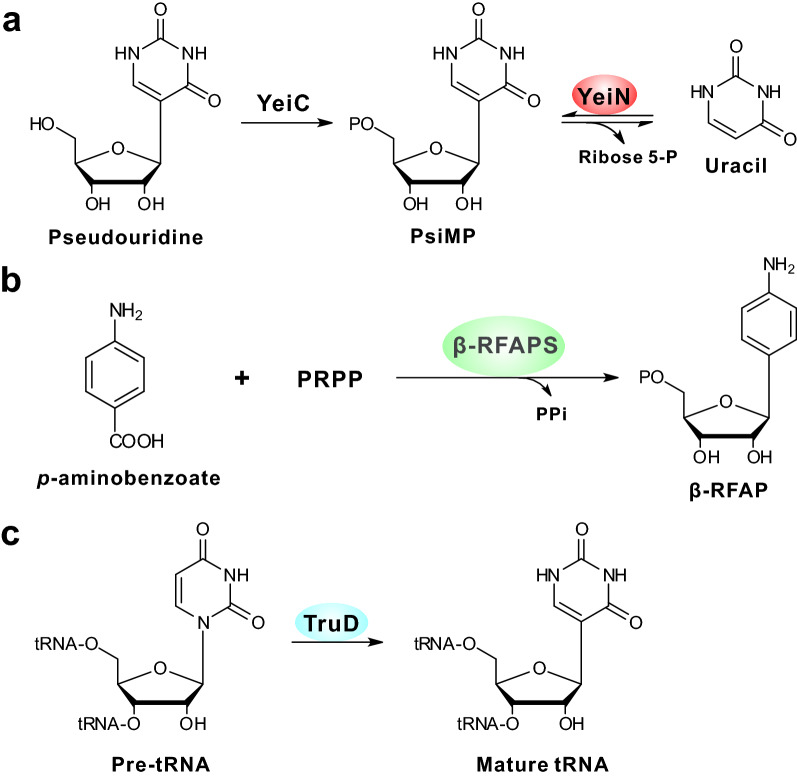
Mechanism of C–C bond formation for related antibiotics. **a** The YeiC phosphorylates pseudouridine to produce PsiMP, and then hydrolyzed to uracil and ribose 5-P by YeiN. **b** β-RFAPS catalyzes the condensation of *p*-aminobenzoate (*p*ABA) and PRPP to β-RFAP. **c** The pseudouridine is generated from isomerization of uridine-tRNA, catalyzed by TruD. YeiC, pseudouridine kinase; ribose 5-P, ribose-5′-Phosphate; β-RFAPS, β-RFAP synthase

Although *C*-nucleoside antibiotics (Fig. 1a–c), including showdomycin (SDM), minimycin (MIN), formycin A (FOR-A), pyrazofurin A (PRF-A), malayamycin A (MAL-A), and pseudouridimycin (PUM), have been demonstrated to be an attractive group of natural products as leads in pharmaceutical development, their biosynthesis is poorly understood and has not been systematically investigated until recently. According to the enzymatic strategies employed for the construction of *C*-glycosidic bonds, the six *C*-nucleoside antibiotics can be divided into three sub-groups (Fig. 1a–c). For the SDM and MIN molecules, C–C bond construction is dependent on a YeiN-like (YeiN, pseudouridine-5′-phosphate glycosidase from *Escherichia coli*) *C*-glycosynthase (Fig. 2a) [5, 6], while FOR-A and PRF-A biosynthesis utilize a β-ribofuranosyl-aminobenzene 5′-phosphate (β-RFAP) synthase-like enzyme to catalyze the *C*-glycoside bond formation (Fig. 2b) [7, 8]. As for the MAL-A and PUM nucleoside antibiotics, a tRNA pseudouridylate synthase (TruD-like) has been identified to be involved in the *N*- to *C*-isomerization of a nucleoside-like intermediate (Fig. 2c) [9, 10].

Several biosynthetic gene clusters (BGCs) of actinobacterial *C*-nucleosides have been recently identified and characterized [6, 7, 11], which, for the first time, has enabled scientists to explore the biosynthetic panorama of them, and has also provided the basis for future *C*-nucleoside-derived drug discovery and development. More than that, the reservoirs of the conserved genes for *C*-nucleosides biosynthesis have been increased with unprecedented speed in the past few years, accordingly, genome mining, as an integrated and powerful strategy, has been conferred with great capacities to revitalize the process for the rapid and rational discovery of more novel *C*-nucleoside molecules with diverse biological activities [12].

In this review, we mainly summarize the recent progress on the biosynthesis of *C*-nucleoside antibiotics. Moreover, we tentatively delineate the perspectives as well on the rapid discovery and rational generation of more *C*-nucleoside antibiotics with utilization of synthetic biology strategies.

## Biosynthesis of the YeiN-like type *C*-nucleoside antibiotics

### Biosynthesis of showdomycin features disassociated NRPS enzymes

Showdomycin (SDM) was isolated from *Streptomyces showdoensis* ATCC 15227 (*S. showdoensis*, likewise, other strains were abbreviated in this paper) in 1964 and was synthesized chemically in 1970 [13–15]. The structure of SDM is shown as 3-β-d-ribofuranosylmaleimide, which possesses a unique maleimide-type ring. The SDM molecule is structurally related to pseudouridine but lacks a –NH group in heterocycle [16]. SDM shows a broad spectrum activity against Gram-positive and Gram-negative bacteria, moreover, it is active against HeLa cells and Ehrlich mouse ascites tumor in vivo by inhibiting DNA and RNA polymerases [16]. Previous feeding studies demonstrated that succinate, fumarate, malate, and acetate are converted to α-ketoglutarate or glutamate via the Krebs cycle in *S. showdoensis,* and α-ketoglutarate is then decarboxylated to give rise to an asymmetrical C4 unit which serves as C-2 to C-5 of maleimide ring as for the ribosyl moiety, it is directly originated from d-ribose [17–19].

**Fig. 3:**
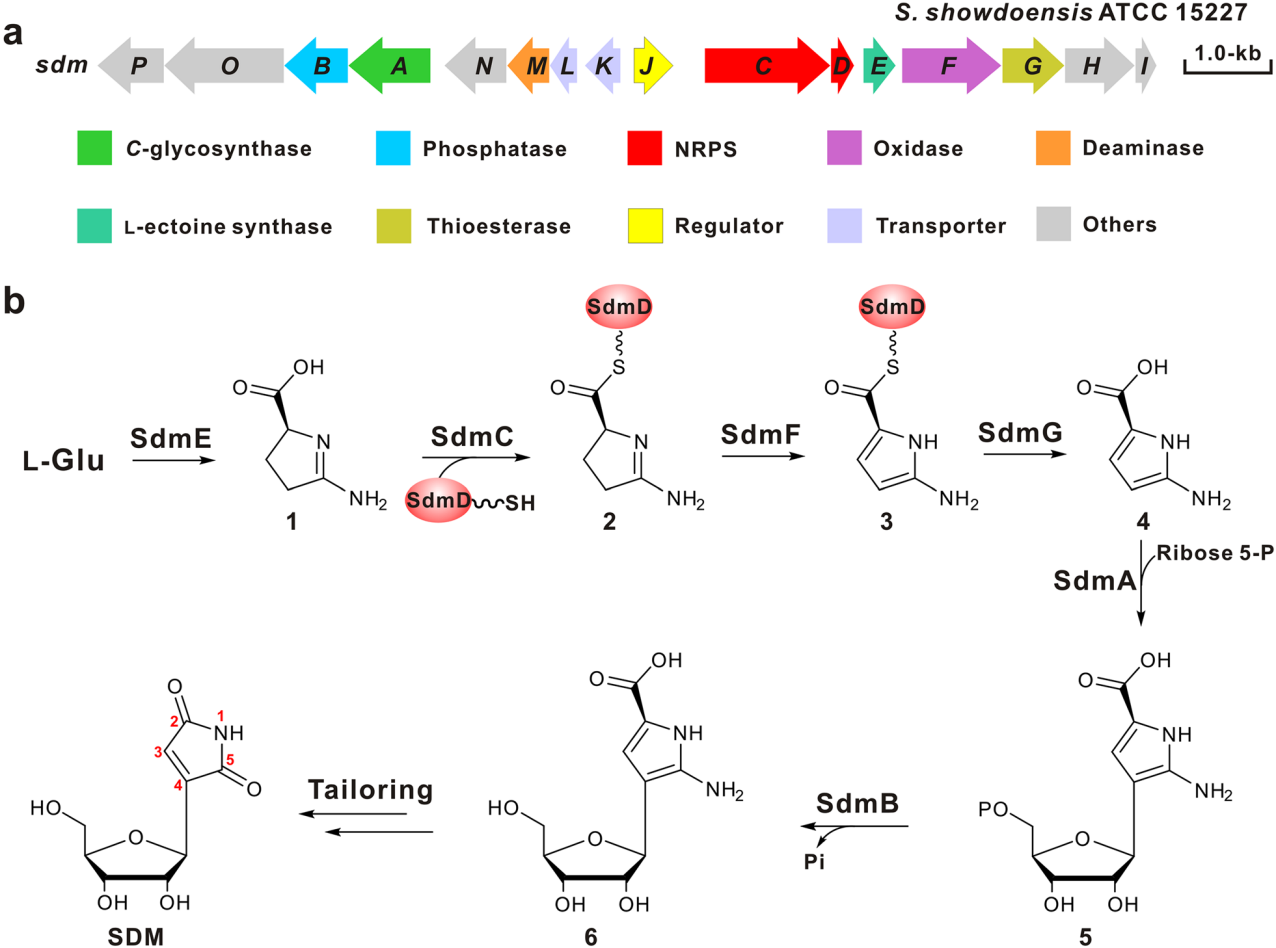
Genetic organization of the SDM gene cluster and its proposed biosynthetic pathway. **a** Genetic organization of the SDM gene cluster. The gene cluster (*sdm*) was identified from *S. showdoensis* ATCC 15227. **b** Proposed biosynthetic pathway to SDM. The gene organization was analyzed by BLAST (https://blast.ncbi.nlm.nih.gov/Blast.cgi), antiSMASH (https://antismash.secondarymetabolites.org/#!/start), and 2ndfind (https://biosyn.nih.go.jp/2ndfind/), and these online programs were used as well for the bioinformatics analysis of other gene clusters and genomes mentioned in this paper

The SDM BGC was identified from the genome of *S. showdoensis* using AlnA (*C*-glycosynthase) and AlnB (phosphatase) from alnumycin pathway as the query sequences, and the SDM BGC is composed of 16 genes and spans a 12.2-kb continuous chromosome region (Fig. 3a, Table 1) [5]. Biosynthesis of the SDM molecule is proposed to be initiated by the cyclase SdmE, which is responsible for the cyclization of the substrate l-glutamine. Then the intermediate **1** (2-amino-1-pyrroline-5-carboxylate) is activated by the nonribosomal peptide synthetases SdmC to produce compound **2**, which is subsequently loaded onto the phosphopantetheine arm of the peptidyl carrier protein SdmD. The tethered intermediate subsequently undergoes successive oxidation and hydrolysis to synthesize **4** [20], afterwards, this intermediate (**4**) is sequentially catalyzed by *C*-glycosynthase SdmA and dephosphatase SdmB to produce the intermediate **6**, which experiences a series of nonenzymatic steps including decarboxylation, deamination, and oxidoreduction to generate the end product SDM (Fig. 3b) [20]. Actually, the proposed SDM biosynthetic pathway has been as yet incompletely understood.

**Table 1:** Summary of the bioactivities and biological targets for the described natural nucleoside antibiotics

Antibiotics	Producing strains	Bioactivities	Biological targets	BGC Accession no
Showdomycin	*S. showdoensis* ATCC 15227	Antibacterial	DNA and RNA polymerases	LAQS00000000^a^
Minimycin	*S. hygroscopicus* JCM 4712	Antibacterial, antitumor	RNA polymerase	MK122964
Formycin A	*Noc. interforma* ATCC 21072 *S. kaniharaensis* ATCC 21070	Antiviral, antibacterial	Adenosine kinase	KY682079 KY705052 MK830994 WBOF00000000^b^
Pyrazofurin A	*S. candidus* NRRL 3601	Antiviral, antitumor	OMP decarboxylase	MH493900 MN305320
Malayamycin A	*S. malaysiensis* DSM 14072 *S. chromofuscus* ATCC 49982	Antiviral, antifungal, anticancer	Unknown	KJ493330 MH537786
Pseudouridimycin	*Streptomyces* sp. ID38640 *S. albus* DSM 40763	Antibacterial	RNA polymerase	MG266907 RCIY01000000^c^

^a^The SDM BGC occupies an approximate 12.2-kb chromosomal region in *S. showdoensis* ATCC 15227

^b^The genome sequence of *S. kaniharaensis* ATCC 21070 is available in GenBank with the accession number WBOF00000000

^c^The PUM BGC from *S. albus* DSM 40763 is available with the accession RCIY00000000

### Biosynthesis of minimycin highlights a NRPS machinery

Minimycin (MIN) (also called oxazinomycin) was produced by both *Streptomyces* sp. and *Pseudomonas* sp. [21–23]. The MIN molecule distinguishes a unique structural system, in which the 1,3-oxazine-2,4-dione ring and the ribosyl sugar are linked via a C–C bond [24]. MIN is structurally similar to pseudouridine, and actually it is prone to being slightly converted to pseudouridine under mild alkaline environment [25]. MIN is active against both Gram-positive and Gram-negative bacteria; moreover, it possesses antitumor activity against transplantable tumors [21, 26]. Recently, MIN is revealed to be able to inhibit RNA polymerase at the polythymidine sequences, whereas the detailed action mechanism of MIN has as yet been unclear [26]. With regards to the biosynthetic origin of MIN, C-6, C-5, and C-4 of the oxazine ring arise from the corresponding C-3, C-4, and C-5 of l-glutamate, and the C-2 of MIN is derived from carbon dioxide, and the ribosyl portion of MIN derives from ribose [27, 28].

**Fig. 4:**
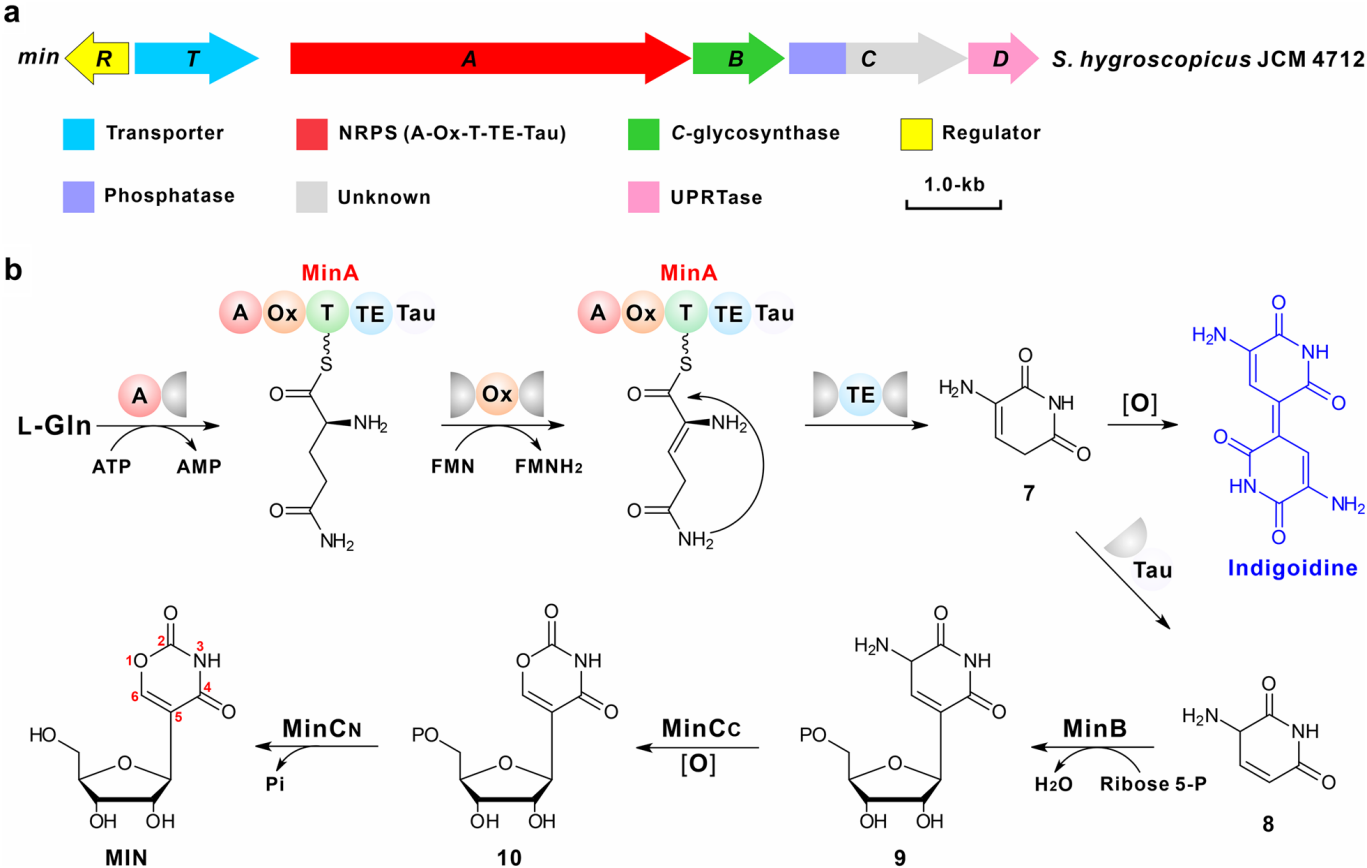
The gene cluster as well as the proposed pathway for MIN biosynthesis. **a** Genetic organization of the MIN gene clusters; the gene cluster was cloned from *S. hygroscopicus* JCM 4712. **b** Proposed biosynthetic pathways to MIN; A, adenylation domain; Ox, oxidase domain; T, thiolation domain; TE, thioester domain; Tau, tautomerase domain

The MIN biosynthetic gene cluster has recently been identified and characterized, and the intact MIN BGC consists of five essential genes involving *minA*, *minB*, *minC*, *minD*, and *minT* (Fig. 4a, Table 1) [6]. MinA (non-ribosomal peptide synthetase, NRPS), MinB (*C*-glycosynthase), and MinC (HAD phosphatase and DUF4243 domain) are confirmed to be responsible for synthesizing the MIN compound [6]. Biosynthesis of the MIN molecule is started by the NRPS enzyme MinA, and the substrate l-glutamine can be specifically selected and activated by adenylation domain (A domain) in the presence of ATP, and then the activated amino acid is tethered on the thiolation domain (T domain). The C_α_-H of the tethered l-glutamine is deprotonated by the conserved active site (Tyr residue) in oxidase domain which initiates the oxidation reaction at C-2 to C-3 positions [29]. The oxidized complex is successively cyclized and released by the thioesterase domain (TE domain) to obtain the intermediate compound **7**, which can be converted to indigoidine by a spontaneous coupling reaction. Simultaneously, this intermediate can also be tautomerized by the tautomerase domain (Tau domain) to generate compound **8**, which is accepted by MinB as substrate for *C-*glyosidic bond construction to form compound **9** [6]. Finally, the intermediate **9** is sequentially catalyzed by the *C*-terminal domain and *N*-terminal domain of MinC (MinC_C_ and MinC_N_, respectively) to synthesize the end molecule MIN, with undergoing an unusual oxidative deamination–recombination reaction associated with the final dephosphorylation (Fig. 4b) [6].

More interestingly, MIN biosynthesis employs an unprecedented self-resistance mechanism. In this resistance system, most of the intracellular compound **10** (MIN monophosphate) was dephosphorylated by MinC_N_ (the *N*-terminal phosphatase domain of MinC) to produce MIN, and most of MIN was transported out of the cell by the transporter MinT, thereby greatly alleviating the toxic effect of intracellular MIN monophosphate. Moreover, MinD (uracil phosphoribosyltransferase) enhances the concentration of in vivo UMP pool to achieve the competition advantage against the relatively poor amount of cellular MIN monophosphate. Accordingly, MinC_N_, MinD, and MinT function together as safeguard enzymes, and collaborate to fulfill the mission of self-resistance during MIN biosynthesis [6].

## Biosynthesis of the β-RFAP synthase-like type *C*-nucleoside molecules

### Formycin A biosynthesis features an unusual pathway for pyrazole ring formation

Formycin A (FOR-A) was first isolated from the fermentation broth of *Nocardia interforma* in 1964 during the searching for antitumor compounds [30]. This antibiotic has also been discovered in culture filtrates of other strains, including *Streptomyces lavendulae* [31], *Streptomyces kaniharaensis* ATCC 21070 (*S. kaniharensis*) [32], and *Streptomyces resistomycificus* NRRL 2290 [7]. Subsequently, FOR-A and coformycin (COF) (an adenosine deaminase inhibitor harboring a 1,3-diazepine ring) were found to be concomitantly produced by these strains [7]. The structure of FOR-A was illustrated and confirmed as 7-amino-3-β-d-ribofuranosyl-1H-pyrazolo-[4,3d] pyrimidine by X-ray crystallography [33–35]. FOR-A, as an adenosine analog targeting adenosine kinase, exhibits significant antitumor activity against *Ehrlich carcinoma* in mice as well as HeLa cells [30], moreover, it exhibits antiviral activity against influenza virus A1 and human immunodeficiency virus type 1 [36, 37]. However, FOR-A has to be phosphorylated at the 5′ position to give the active molecule FOR-A monophosphate, which is able to block the de novo biosynthesis of purine and pyrimidine nucleotides [38]. Structurally, ribosyl moiety of FOR-A is derived from ribose of phosphoribosyl pyrophosphate (PRPP) [39], and the N-3 and N-8 atoms of FOR-A originate from the ε-amino group of l-lysine (l-Lys) [40]. Moreover, the C-6, C-5, C-4, and C-9 carbons of FOR-A are derived from the C-1, C-2, C-3, and C-4 carbons of glutamate [41].

**Fig. 5:**
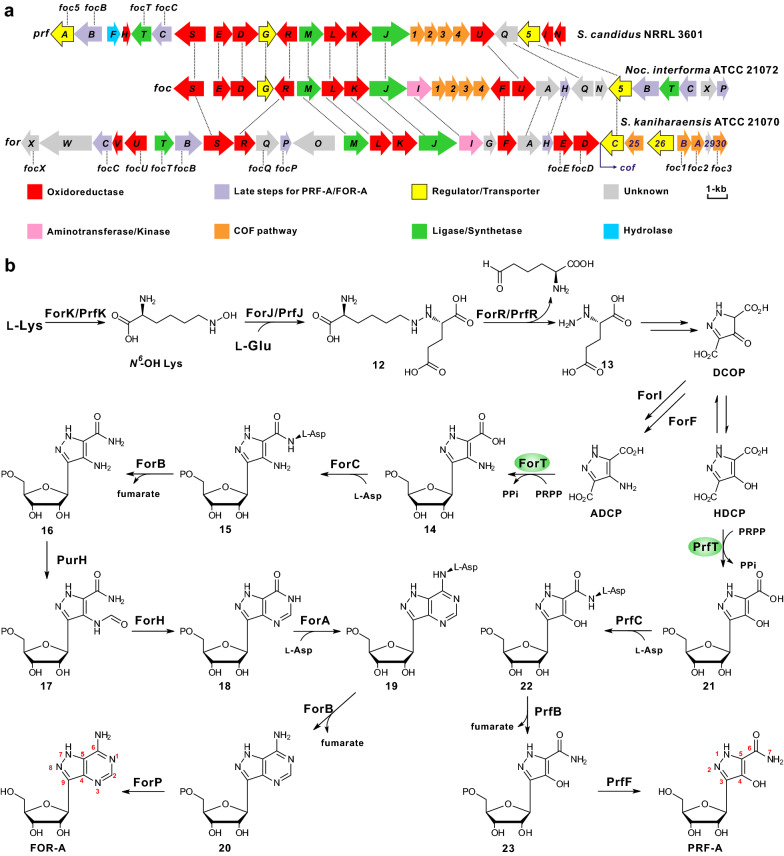
The gene clusters and corresponding pathways for FOR-A and PRF-A biosynthesis. **a** Genetic organization of the PRF-A and FOR-A gene clusters. The PRF-A gene cluster (*prf*) was from *S. candidus* NRRL 3601, and the FOR-A and COF gene cluster was from *Nocardia interforma* ATCC 21072 and from *S. kaniharaensis* ATCC 21070, respectively. **b** Proposed pathways to FOR-A and PRF-A. They share identical steps at the early stage prior to the construction of *C*-nucleoside scaffolds. HDCP, 4-hydroxy-3,5-dicarboxypyrazole; DCOP, 3,5-dicarboxy-4-oxo-4,5-dihydropyrazole; ADCP, 4-amino-3,5-dicarboxypyrazole

Recent investigations have illuminated that the pyrazolopyrimidine moiety of FOR-A was formed in a similar manner to that of adenosine [42], which is actually assembled by the Pur enzymes, including adenylosuccinate synthetase PurA, adenylosuccinate lyase PurB*,* SAICAR (phosphoribosylaminoimidazole-succinocarboxamide) synthetase PurC, and AICAR (5-aminoimidazole-4-carboxamide ribonucleotide) transformylase PurH. In a recent study, two sets of Pur enzymes were identified in genome of *S. kaniharaensis*, and the second set of Pur-like enzymes (the counterpart for enzymes) is undoubtedly involved in the biosynthesis of FOR-A [43]. A draft genome sequence of *S. kaniharaensis* was assembled from 19 polished contigs with an N50 contig length of 6,153,183 bp [44]. The gene cluster from *S. kaniharaensis* is composed of 30 genes, and occupies a ca. 37.0-kb continuous chromosomal region, while the counterpart *foc* gene cluster (26 genes) from *Nocardia interforma* ATCC 21072 is ca. 29.4-kb in size (Fig. 5a, Table 1) [7]. More interestingly, the antibiotic pair COF-FOR-A also employs an unusual but general protector-protégé strategy; i.e., COF may protect FOR-A from deamination by the housekeeping adenosine deaminase [45].

Biosynthesis of FOR-A is initiated by ForK (l-Lys *N*^*6*^-monooxygenase) with hydroxylation of l-Lys to form *N*^*6*^-OH l-Lys [7]. Subsequently, *N*^*6*^-OH l-Lys and l-glutamate are catalyzed by methionine-tRNA ligase ForJ, with constructing the unusual N–N bond of compound **11** between the α-amino group of Glu and the ε-amino group of Lys. Then, the amino acid oxidase PrfR, catalyzes the oxidative cleavage of the C–N bond of **11** to form compound **12** [46], which then experiences sequential reactions to build 3,5-dicarboxy-4-oxo-4,5-dihydropyrazole (DCOP). After successive reactions by ForI (aminotransferase) and ForF (phosphoglycerate dehydrogenase), DCOP is catalyzed to form ADCP (4-amino-3,5-dicarboxypyrazole). ForT (β-ribofuranosyl-aminobenzene 5′-phosphate synthase-like enzymes) is responsible for the condensation of PRPP and ADCP to form a carboxyaminopyrazole ribonucleotide via a similar mechanism employed in methanopterin biosynthesis [7, 8]. After that, **13** is converted to **19** through sequential enzymatic steps closely identical to those in the purine biosynthetic pathway of primary metabolism [42]. After final dephosphorylation, compound **19** is catalyzed to form the end product FOR-A (Fig. 5b). The crystal structures and mechanisms of several enzymes, involving the PLP-dependent transaminase ForI [47] and the *C*-glycoside synthase ForT [48], have recently been solved and elucidated, which will provide the solid basis for the further engineering of FOR biosynthesis towards improved production/activities.

### The biosynthetic pathways of pyrazofurin A and formycin A are overlapped

Pyrazofurin (PRF-A) was isolated from the cultures of *Streptomyces candidus* NRRL 3601 (*S. candidus*) in 1969, and its structure is 4-hydroxy-3-β-d-ribofuranosyl-pyrazole-5-carboxamide as determined by a combination of spectroscopy and chemical degradation (Fig. 1) [49]. Both PRF-A and FOR-A feature pyrazole-derived nucleobases, either imidazolopyrimidine (for PRF-A) or pyrazolopyrimidine (for FOR-A), which are obviously different from the typical nucleosides [50]. Recently, PRF-A and formycin B (a deaminated product of FOR-A) were synthesized with seven steps by employing a sydnone riboside as common intermediate [51].

In the past decades, PRF-A has been of great research interests in medicinal chemistry due to its remarkable activities and application potential [52]. PRF-A harbors notable antiviral and antitumor activities, moreover, this molecule indicates antineoplastic activity in rats and is also active to a relatively broad range of tumors, including Walker carcinosarcoma, plasma cell myeloma, and various types of lymphosarcoma and breast carcinoma [53, 54]. Mechanistically, PRF-A targets orotidine-5′-monophosphate (OMP) decarboxylase with rendering inhibition of pyrimidine biosynthesis, simultaneously, this *C*-nucleoside molecule is also a potent inhibitor of 5-aminoimidazole-4-carboxamide ribotide (AICAR) transformylase of the purine pathway [55, 56]. Initially, PRF-A has been tentatively developed as a potential anticancer drug, unfortunately, unsatisfactory results were emerged in the phase I clinical trials because of its toxicity to human cells [57, 58]. Afterwards, development of PRF-A as a potential antitumor drug has gradually faded. However, a recent study with exciting result that PRF-A was demonstrated to be effective against SARS-associated coronaviruses, making it potential to develop antiviral interventions of the global pandemic COVID-19 [59].

Concerning the biosynthetic origin of PRF-A, C-3 to C-6 unit of PRF-A is derived from C-4 to C-1 of glutamate and/or α-ketoglutarate on the basis of previous isotope feeding experiments [60], and the gene cluster of PRF-A was recently identified from *S. candidus* using FocJ (an annotated methionine-tRNA ligase) as the query sequence [7]. The *prf* gene cluster consists of 24 genes and occupies a continuous 27.1-kb chromosomal region (Fig. 5a, Table 1) [7]. More surprisingly, the genes for COF biosynthesis were also revealed to be existed in *prf* gene cluster, however, no related product was identified from cultures in recent studies [61].

The PRF-A molecule is structurally similar to FOR-A, and the pathways of them are actually overlapped. The biosynthetic pathway of PRF-A, resembling that of FOR-A, also contains a pyrazole-related intermediate DCOP, which spontaneously undergoes keto-enol isomerization to form 4-hydroxy-3,5-dicarboxypyrazole (HDCP). After that, HDCP and PRPP are catalyzed by PrfT (β-ribofuranosyl-aminobenzene 5′-phosphate synthase-like enzymes) to form C–C bond of compound **20** [7]**.** Finally, PrfC (SAICAR synthetase) and PrfB (a putative adenylsuccinate lyase) are shown to catalyze amidation of compound **20** to generate pyrazofurin 5′-phosphate (compound **21**). Subsequently, this intermediate is under catalysis of PrfB with leaving of a fumarate. After removal of phosphate group, biosynthesis of the end molecule PRF-A is accomplished (Fig. 5b) [8].

## Biosynthesis of the TruD-like type *C*-nucleoside natural products

### Biosynthesis of malayamycin A resembles polyoxin in the backbone construction

Malayamycin A (MAL-A), was originally isolated from broth of the soil bacterium *Streptomyces malaysiensis* DSM 14702 (*S. malaysiensis*) by a group at the Syngenta Crop Protection laboratories in Jealott’s Hill, U.K [62], and the MAL-A molecule contains a bicyclic perhydrofuropyran “sugar” moiety rather than the more commonly encountered monocyclic pentofuranosyl or hexopyranosyl core [63]. MAL exhibits potent antiviral, antifungal, and anticancer bioactivity, moreover, MAL displays broad spectrum activity against phytopathogenic fungi in the greenhouse, for instance, MAL inhibits sporulation of *Stagonospora nodorum* (Berk) Castell & Germano, the cause of *Stagonospora nodorum* blotch of wheat [64], while the detailed action mechanism for MAL has still remained obscure.

**Fig. 6:**
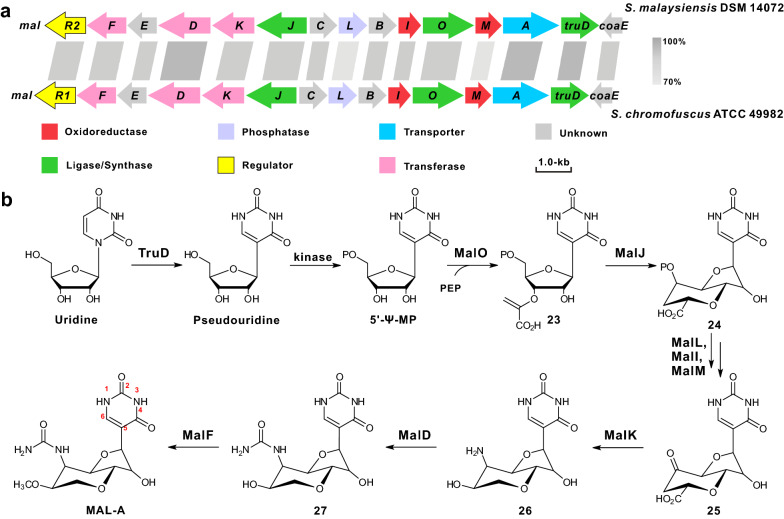
The gene clusters and proposed pathway for MAL-A biosynthesis. **a** Organization of the MAL-A gene clusters (*mal*), which is independently identified from *S. chromofuscus* ATCC 49982 and *S. malaysiensis* DSM 14072. **b** The proposed biosynthetic pathway to MAL-A

The discovery and characterization of the biosynthetic pathway to MAL-A was previously reported using genome mining of near-identical clusters both from the known producer *S. malaysiensis* and from *S. chromofuscus* (Fig. 6a, Table 1) [10]. A strong candidate to be the MAL-A biosynthetic gene cluster, comprises 20 predicted open reading frames. The biosynthesis of MAL-A may utilize uridine as a starter substrate by pseudouridine synthase TruD to generate pseudouridine [10], which is susceptible to be phosphorylated to produce 5′-pseudouridine monophosphate (5′-Ψ-MP). After that, 5′-Ψ-MP was catalyzed by MalO (enoylpyruvyl transferase) acting almost exclusively on 5′-Ψ-MP to yield compound **23**. Then, the radical SAM enzyme MalJ is proposed to catalyze C–C bond formation utilizing a previously-characterized strategy in nikkomycin/polyoxin biosynthesis to form compound **24**, which is then catalyzed by enzymes containing phosphatase MalL, 2-oxoglutarate-Fe (II)-dependent oxygenases MalM and MalI, and aminotransferase MalK to build compound **26** [10]. After the final tailoring enzymatic steps by the carbamoyltransferase MalD and the *O*-methyltransferase MalF, biosynthesis of the end *C*-nucleoside molecule MAL-A is accomplished (Fig. 6b) [10].

### Biosynthesis of the bacterial RNA polymerase-pseudouridimycin

In 2017, it has been reported the identification and complete characterization of pseudouridimycin (PUM), a new *C*-nucleoside antibiotic from *Streptomyces* sp. ID38640, by a team of scientists from Rutgers University and from Naicons in collaboration with the university of Milan and the university of Bonn [65]. PUM is a peptidyl nucleoside, and is composed of a cored 6′-amino-pseudouridine and a formamidinylated, *N*-hydroxylated glycine–glutamine side chain [66]. PUM covers a broad antibacterial spectrum, including some drug-sensitive and drug-resistant strains, which is due to its high affinity and unusual mechanism with its target, RNA polymerase (RNAP) [67]. PUM can mimic the substrate of RNAP, nucleoside-triphosphate (NTP), then accurately occupies and tightly binds the NTP addition site on RNAP by preventing the addition of NTP [68]. Another exciting feature of PUM is its selectivity: it can selectively inhibit the bacterial RNAP polymerase (bRNAP) in vitro rather than human RNA polymerase (hRNAP) [69, 70].

**Fig. 7:**
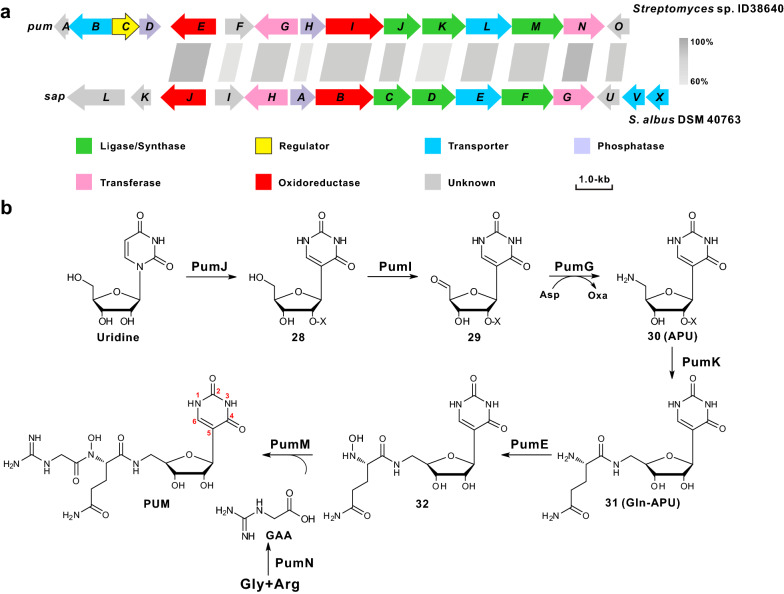
Genetic organization of the PUM gene cluster and the proposed pathway to PUM. **a** Genetic organization of the PUM gene cluster. The gene cluster (*pum*) was from *Streptomyces* sp. ID38640. The *sap* gene cluster was from *Streptomyces albus* DSM 40763, show high homology to *pum* gene cluster. **b** Proposed biosynthetic pathways to PUM. APU, 5′-amino pseudouridine; Glu-APU, Glutamine-APU; GAA, guanidino acetic acid. X = H or PO_3_H_2_

The PUM biosynthetic gene cluster has been identified in *Streptomyces* sp. ID38640, and the *pum* cluster is likely to encompass a genomic segment of about 20-kb consisting of 15 genes (designated *puma*–*pumO*) associated with PUM biosynthesis, export, and regulation (Fig. 7a, Table 1) [9]. The knock-out mutant strains were correspondingly able to accumulate the specialized intermediates, including pseudouridine (PU), 5′-amino pseudouridine (APU), Gln-APU, *N*-hydroxy-Gln-APU guanidinoacetic acid (GAA) [9]. Recently, the minimal PUM BGC was generated and expressed in the heterologous host *Streptomyces coelicolor* M1146 under control of strong promotors including *pumB* and *pumD–N* [71, 72]. Besides that, all core genes involved in the formation PUM could be identified in *Streptomyces albus* DSM 40763 (*S. albus*), a previously reported strain capable of producing the *C*-nucleoside strepturidin, which leads to the structural revision of the antibiotic as PUM. Actually, the core *sap* (the gene cluster of PUM from *S. albus*) and *pum* gene clusters share both significant homology and the almost-identical genetic organization (Fig. 7a) [73]. Based on in silico analysis, genetic, and feeding experiments of precursors or intermediates, the complete PUM biosynthetic pathway was tentatively proposed as indicated in Fig. 7b [71].

In the proposed pathway to PUM, the initial substrate uridine is isomerized to *C*-nucleoside intermediate **28** by the tRNA pseudouridine synthase PumJ (TruD-like pseudouridylate synthase), and the cored nucleoside region is successively modified by oxidoreductase PumI and aminotransferase PumG to generate APU (intermediate **30**) [9]. During the early steps, the kinase PumH can recognize uridine, **28** or **29** as substrates and phosphorylation likely occurs at the C-2′ position, similarly to the recently-illustrated reaction by PolQ2 (the PumH homolog, a kinase in the biosynthesis of polyoxin) [74]. Subsequently, the phosphate group is removed by PumD or by a housekeeping phosphatase. The compound **30** is then condensed to glutamine, a reaction catalyzed by the amide ligase PumK to yield the intermediate Gln-APU (**31**). After that, the intermediate **31** is *N*-hydroxylated by the oxidoreductase PumE to generate **32**. In a parallel way, another intermediate GAA is produced from glycine and arginine by the amidinotransferase PumN, and the GAA molecule is added to compound **32** by PumM leading to the final compound, PUM, which is transported out of the cell by PumL (transporter) [71]. More recently, PumF has been identified as a positive regulator by controlling the transcription of the *pumK–N* unit [71].

## Conclusions and perspectives

Over the past decades, *C*-nucleoside antibiotics have distinguished themselves by diverse biological activities and unusual structural features, and they, as spotlight molecules, have attracted increasing research interests for pharmaceutical applications by medicinal chemists and pharmacologists. The present review mainly summarized the recent progress on the biosynthesis of *C*-nucleoside antibiotics, which may enlighten us to explore the future opportunities of these antibiotics for clinical applications. Furthermore, the technological innovations and conceptual breakthroughs have emerged with an accelerated rhythm, which would promise a renaissance in the future for the discovery of novel *C*-nucleoside antibiotics.

**Fig. 8:**
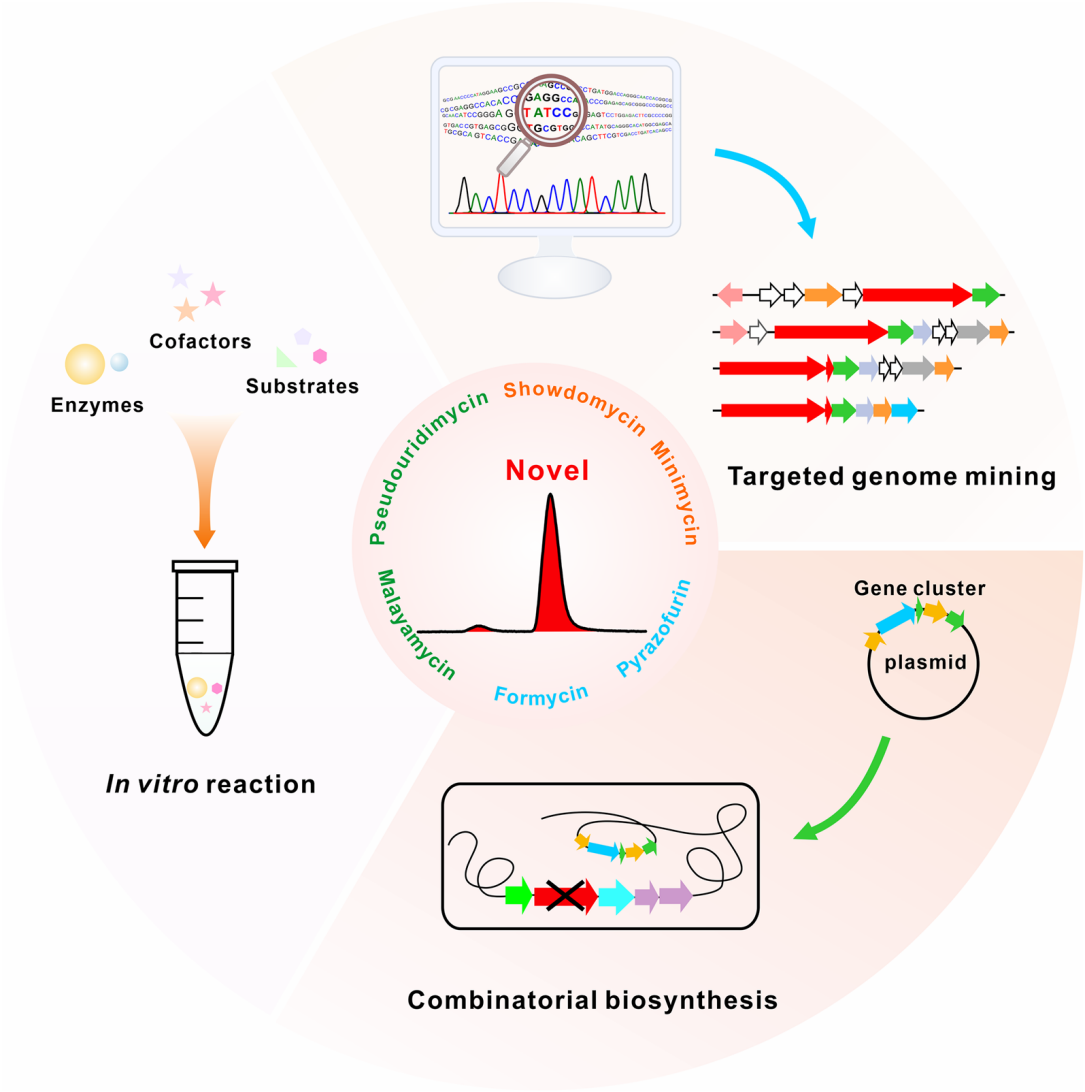
Strategies for rapidly and rationally accessing novel *C*-nucleoside antibiotics. As shown in this figure, three strategies, involving targeted genome mining, combinatorial biosynthesis, and in vitro reaction, were delineated for rapidly accessing chemical diversities of *C*-nucleoside antibiotics in the future

With the considerable advances in genome sequencing technology, particularly in the past decade, enormous reservoirs of microbial genomes have become explosively available [75]. Scientists have been enabled with the unprecedented power, for the first time, to create chemical diversities of more novel *C*-nucleoside antibiotics towards enhanced/improved properties. We could envision that more BGCs related to *C*-nucleoside antibiotics will be rapidly and rationally accessed by targeted genome mining with great assistance of big data and artificial intelligence (Fig. 8). Moreover, molecular phylogeny of key biosynthetic genes present in their gene clusters can be used as a pre-screening method to prioritize gene clusters for detailed biosynthetic analysis and molecule expression studies from a large pool of sequenced or un-sequenced microorganisms [76]. Besides, modifying the promoters, manipulating the regulatory genes, and activating the silent gene clusters by the CRISPR/Cas9 (derived) technologies, confer scientists with enormous opportunities for further targeted discovery of new *C*-nucleoside compounds [77].

Furthermore, we do expect, with accessing more BGCs coding for *C*-nucleoside molecules, that scientists will be provided with great possibilities to generate *C*-nucleoside diversities by more extensive synthetic biology-based strategies, including precursor-directed biosynthesis, mutasynthesis, combinatorial biosynthesis, and other biosynthetic manipulations [10]. Also, the actinobacteria chassis can be further engineered by feeding intermediates to the knockout mutant for increasing diversities of the final antibiotic products. Indeed, it would be of great expectation for scientists to develop an in vitro reaction platform for high-efficient generation of *C*-nucleoside analogs (Fig. 8). We can anticipate, with more novel enzyme tools for *C*-nucleoside biosynthetic pathway illustrated and available, that opportunities to discover and generate improved *C*-nucleoside antibiotics in the future will be accelerated in a more rational and economical way [78].

In the past, clinical application of the *C*-nucleoside antibiotics has been severely hampered accounting for their potential toxicities and limited diversities, nevertheless, *C*-nucleoside related drugs (such as Remdesivir) with potent clinical efficacies have been artificially created and approved to combat epidemic diseases [79]. In this respect, more combined and intensive efforts should be paid in the future for targeted discovery of more *C*-nucleoside molecules from natural reservoir of microbial genomes under the guidance of multiple strategies.

## Data Availability

The datasets supporting the review are included within the article.

## Abbreviations

SDM: Showdomycin
MIN: Minimycin
FOR-A: Formycin A
PRF-A: Pyrazofurin A
MAL-A: Malayamycin A
PUM: Pseudouridimycin
Ψ: Pseudouridine
BGC: Biosynthetic gene cluster
NRPS: Non-ribosomal peptide synthetase
A: Adenylation domain
Ox: Oxidase domain
T: Thiolation domain
TE: Thioesterase domain
Tau: Tautomerase domain
COF: Coformycin
PRPP: Phosphoribosyl pyrophosphate
l-Lys: l-lysine
DCOP: 3,5-Dicarboxy-4-oxo-4,5-dihydropyrazole
ADCP: 4-Amino-3,5-dicarboxypyrazole
HDCP: 4-Hydroxy-3,5-dicarboxypyrazole
β-RFAP: β-Ribofuranosyl-aminobenzene 5′-phosphate
*p*ABA: *p*-Aminobenzoate
SAICAR: Phosphoribosylaminoimidazole-succinocarboxamide
RNAP: RNA polymerase
NTP: Nucleoside-triphosphate
bRNAP: Bacterial RNAP polymerase
hRNAP: Human RNA polymerase
PU: Pseudouridine
APU: 5′-Amino pseudouridine
Glu-APU: Glutamine-APU
GAA: Guanidinoacetic acid
